# Circulating alpha-1 antitrypsin and its c-terminal peptides differentiate bacterial from viral community-acquired pneumonia

**DOI:** 10.1186/s12967-026-08651-8

**Published:** 2026-07-20

**Authors:** Milad Pashai Fakhri, Friedemann R. Börner, Julia Held, Kokilavani Sivaraman, Jan Fuge, Jan Rupp, Grit Barten-Neiner, Mathias W. Pletz, Martin Witzenrath, Gernot Rohde, Jessica Rademacher, Christopher Alexander Hinze, Sabina Janciauskiene, CAPNETZ Study Group

**Affiliations:** 1 Department of Respiratory Medicine and Infectious Diseases, Hannover Medical School, Carl-Neuberg-Str. 1, 30625 Hannover, Germany; 2 Biomedical Research in Endstage and Obstructive Lung Disease Hannover (BREATH), Member of the German Center for Lung Research (DZL), Hannover, Germany; 3 Institute of Clinical Chemistry and Laboratory Diagnostics, University Hospital Jena, Friedrich Schiller University Jena, Jena, Germany; 4 CAPNETZ STIFTUNG, Hannover, Germany; 5 Department of Infectious Diseases and Microbiology, University Hospital Schleswig-Holstein, Lübeck, Germany; 6 Institute of Infectious Diseases and Infection Control, University Hospital, Jena, Germany; 7 Department of Infectious Diseases, Respiratory Medicine and Critical Care, Charité-Universitätsmedizin Berlin, Berlin, Germany; 8 German Center for Lung Research (DZL), Giessen, Germany; 9 Department of Respiratory, Intensive Care and Sleep Medicine, Philipps-University Marburg, Marburg, Germany; 10 Institute for Experimental Virology, TWINCORE Centre for Experimental and Clinical Infection Research, Hannover, Germany

**Keywords:** Community-acquired pneumonia, Viral, Bacterial, Alpha-1 antitrypsin, C-terminal peptides

## Abstract

**Background:**

Distinguishing bacterial from viral community-acquired pneumonia (CAP) remains a major clinical challenge, often leading to inappropriate antimicrobial use. Alpha-1 antitrypsin (AAT) is an acute-phase protein that regulates neutrophil protease activity and is cleaved during inflammation, generating bioactive peptides. We investigated whether circulating AAT and its peptides could discriminate bacterial from viral CAP.

**Methods:**

Serum samples were obtained from 81 prospectively enrolled adults with CAP (bacterial, *n* = 36; viral, *n* = 45) at hospital admission (day 0) and day 3. AAT concentrations were measured by ELISA, and nine AAT-derived C-terminal peptides were quantified by LC-MS/MS. Associations with CAP etiology were assessed using multivariable logistic regression and receiver operating characteristic (ROC) analyses.

**Results:**

AAT concentrations were significantly higher in bacterial than viral CAP at both admission (*p* = 0.006) and day 3 (*p* < 0.001) and remained independently associated with bacterial etiology after adjustment for clinical covariates, whereas C-reactive protein (CRP) did not. A predictive model combining AAT, age, and leukocyte count demonstrated the highest discriminatory performance (AUC = 0.803). Four of nine analyzed peptides (C36, C37, C40, and C42) were consistently detectable. C37 levels were higher in bacterial CAP at admission (*p* = 0.010). C36 showed a similar trend but declined from day 0 to day 3 (*p* = 0.006). In contrast, C40 levels increased in viral CAP (*p* = 0.017), resulting in a higher C40/AAT ratio at admission compared with bacterial CAP (*p* = 0.014). Correlations between AAT, peptides, and inflammatory markers were observed in bacterial but not in viral CAP, indicating distinct patterns of AAT processing.

**Conclusions:**

Circulating AAT independently discriminates bacterial from viral CAP and, when combined with age and leukocyte count, show improved discriminatory performance compared with CRP-based models. Distinct patterns of AAT-derived peptides suggest etiology-specific proteolytic processing and merit further evaluation as markers for differentiating bacterial and viral CAP.

**Supplementary Information:**

The online version contains supplementary material available at 10.1186/s12967-026-08651-8.

## Introduction

Community-acquired pneumonia (CAP) is an infection of the lung parenchyma acquired outside of hospital or long-term care settings [1]. Despite remaining a leading cause of morbidity and mortality worldwide, particularly among elderly individuals and patients with comorbidities, its diagnosis remains challenging [2]. The most common type of CAP is bacterial pneumonia, which can be caused by *Streptococcus pneumoniae*, *Haemophilus influenzae* and *Mycoplasma pneumoniae*. According to studies conducted in Europe, this is followed by bacterial and viral coinfection, and purely viral infections [3–6]. However, pneumonia can also result from fungal, or mycobacterial infections [7]. Furthermore, a range of non-infectious conditions may present with comparable clinical and radiological features, including pulmonary edema, lung cancer, acute respiratory distress syndrome, and interstitial lung diseases such as cryptogenic organizing pneumonia, eosinophilic pneumonia, drug-induced pneumonitis, and vasculitis [8]. Therefore, despite advances in microbiological diagnostics, distinguishing bacterial from viral CAP at hospital admission remains challenging, often leading to empirical antibiotic use and highlighting the need for improved biomarkers that reflect underlying disease mechanisms [9, 10].

Bacterial CAP is typically associated with intense neutrophil recruitment and high protease activity, whereas viral CAP is more often characterized by lymphocyte-driven and interferon-mediated responses [11, 12]. Increased neutrophilic inflammation and protease release are hallmarks of host defense but can also contribute to tissue injury if not tightly controlled [13]. In this protease-rich inflammatory environment, alpha-1 antitrypsin (AAT) functions as an acute-phase protein that protects the lung from protease-mediated tissue injury, primarily through inhibition of neutrophil elastase [14]. Circulating AAT levels increase during CAP [7] and have been reported to be higher in bacterial than viral pneumonia [15], particularly in gram-positive bacterial infections [16].

Upon protease inhibition, AAT undergoes proteolytic cleavage, primarily within its reactive center loop, generating a range of C-terminal peptides. While serine proteases such as neutrophil elastase initiate this process, additional cleavage by non-cognate proteases, including matrix metalloproteinases, can further diversify the resulting peptide repertoire [17]. Increasing evidence suggests that these peptides are not merely degradation products but possess biological activities, including modulation of immune responses, protease activity, and cell signaling [18]. Several AAT-derived peptides have been reported to exhibit immunomodulatory, anti-inflammatory, and antiviral properties, including inhibition of viral entry by VIRIP [19, 20]. Distinct AAT peptide signatures have been observed in bacterial sepsis of pulmonary origin and severe COVID-19, suggesting that AAT proteolytic processing may differ according to infectious etiology [21]. Like bioactive fragments generated through proteolytic processing of our endogenous AAT protein, plant-derived compounds have also been reported to exhibit immunomodulatory and antioxidant properties. The in vivo activity of wound healing of pistachio hull extract (*Pistacia vera L*.) was found to be very significant in rat excision wounds, implying the potential for therapeutic treatment by using the natural extracts of plant products to modify the inflammatory and wound-healing processes [22]. Selenium nanoparticles from biosynthesis using the peels of *Solanum tuberosum* also showed high levels of antioxidant and cytoprotective abilities [23]. Moreover, other antioxidant plant extracts rich in phenolic compounds, like the ones from *Myrtus communis*, also showed strong antioxidant capacities to promote inflammatory resolution [24].

The presence and clinical relevance of AAT-derived peptides in human pneumonia remain unknown. It is therefore of interest to determine whether AAT and its derived peptides can distinguish bacterial from viral CAP and add diagnostic value beyond established biomarkers such as CRP. In this study, we quantified serum AAT and AAT-derived C-terminal peptides in patients with bacterial and viral CAP at hospital admission (day 0) and day 3 and evaluated their performance as biomarkers for CAP etiology discrimination, alone and in combination with clinical parameters.

## Materials and methods

### Patient cohort

Between 2025 and 2026, serum samples were prospectively collected from 41 adults hospitalized with CAP at Hannover Medical School, including 36 patients with bacterial CAP and 5 patients with viral CAP. Samples were obtained at hospital admission (day 0, prior to initiation of antibiotic therapy) and on day 3 of hospitalization. To increase the number of viral CAP cases, serum samples from an additional 40 patients with exclusively viral CAP, collected at corresponding time points, were obtained through the CAPNETZ study group. In total, the study included 81 patients (36 bacterial and 45 viral CAP). The study was approved by the Ethics Committee of Hannover Medical School (approval no. 11910-BO-S-2025). Samples obtained through CAPNETZ were covered by separate ethics approval from Hannover Medical School (approval no. 301–2008). Written informed consent was obtained from all participants.

CAP was defined as the presence of a new pulmonary infiltrate on chest imaging together with compatible clinical features, including cough, fever, dyspnea, or abnormal auscultatory findings [25]. Additional symptoms considered during diagnosis included myalgia, arthralgia, headache, gastrointestinal symptoms, circulatory disturbances, and, particularly in older patients, neurological manifestations. Patients younger than 18 years of age or unable to provide informed consent were excluded.

Etiological classification was based on a consistent framework applied to all patients, integrating microbiological findings with clinical and radiological information. Viral CAP was defined by PCR-based detection of a respiratory viral pathogen in respiratory samples in the absence of a bacterial pathogen. Bacterial CAP was defined either by identification of a bacterial pathogen or, when viral testing remained negative, by a clinical presentation consistent with bacterial pneumonia. In the latter case, the assessment considered focal consolidation on chest imaging, elevated inflammatory markers, and compatible clinical features such as purulent sputum production or, where available, neutrophil predominance in the peripheral blood count or in respiratory samples. This structured approach ensured that each case was assigned according to uniform criteria while preserving the clinical judgment inherent to routine CAP diagnosis.

Disease severity was assessed using the CRB-65 score. Oxygen supplementation during hospitalization was recorded as a marker of clinical severity. Comorbidities, including cardiovascular disease, diabetes, chronic obstructive pulmonary disease, and malignancy, were extracted from medical records (Table 1). Length of hospital stay was defined as the number of days from admission to discharge and duration of therapy was defined as the total number of days of anti-infective therapy administered during hospitalization.

### Sample handling and storage

Serum samples collected prospectively at Hannover Medical School were aliquoted and frozen at −20 °C immediately after processing and were not thawed prior to biomarker analysis. Serum samples of the viral CAP cohort were obtained from the CAPNETZ biobank, where they had been stored at -80 °C under standardized biobanking conditions without intermediate freeze–thaw cycles until analysis (median storage duration 3.5 years, range 1.3–8.7 years). AAT is a highly abundant and stable plasma acute-phase protein that remains stable during long-term storage at −20 °C and −60 °C, with preserved protein integrity and inhibitory activity (100 ±20% of acceptance criteria) [26]. C-terminal AAT peptides are similarly stable, with ≤ ±10% deviation after repeated freeze–thaw cycles and extended room-temperature incubation compared with fresh samples [27, 28].

### Serum AAT analysis by enzyme linked immunosorbent assay (ELISA)

Serum concentrations of AAT were determined using ELISA kit (Cat. -No. DY1268, R&D System, Minneapolis, MN, USA; assay detection range: 125–8000 pg/ml) according to the manufacturer’s instructions. Absorbance was measured at 450 nm with a correction wavelength at 540 nm using a microplate reader (Tecan Infinite M200, Männedorf, Switzerland), and concentrations were calculated based on standard curves.

### Serum analysis of C-terminal peptides of AAT

Serum peptide quantification was performed using ultra-high performance liquid chromatography–tandem mass spectrometry (LC-MS/MS). The quantification method was validated according to common regulations with linear working ranges from 0.01 µM to 1.5 µM that were optimized according to AAT peptide concentrations in healthy and critically ill patients. The lower limit of quantification (LLOQ) was set to 0.01 µM for C-terminal peptides C22, C37, C39, C43, C44 and C45; 0.025 µM for C36 and C42; or 0.0036 µM for C40, as previously published [27, 29, 30]. Peptides could be detected below these thresholds, but values below the corresponding LLOQs were set to these values. The mean accuracy and precisions (within- and between-run repeatability) were within recommended ranges and not bigger than ± 15% and ± 20% of the LLOQ, respectively. LC-MS/MS data processing was done with Analyst Software (version 1.6.2 and 1.7.1). Peak integration was reviewed individually and if applicable analyte peak areas were normalized to the peak area of their respective internal standards. Concentrations were calculated from a quadratic fit standard curve with 1/x*x weighting. All analytes with more than 20% uncertain values (not detected or below LLOQ) were excluded from further analysis [27].

### Statistical analysis

Statistical analyses included Mann Whitney U tests for group comparisons, Wilcoxon signed rank tests for the assessment of temporal changes, and Spearman correlation analyses for evaluation of associations between variables. Both univariate and multivariate logistic regression models were performed, and corresponding receiver operating characteristic (ROC) curves with calculation of the area under the curve (AUC) were derived. C-peptide/ AAT ratios were calculated by converting AAT concentrations from µg/mL to µM using a conversion factor of 0.0192, ensuring consistent units with the C-peptides. All ratios were subsequently multiplied by a factor of 1000 to improve numerical scaling and facilitate data presentation. All analyses were conducted using R (version R 4.5.3). Formal blinding of laboratory personnel to clinical and microbiological data was not performed. All biomarker measurements were conducted using standardized, quantitative assays with objective, instrument-based readouts, including ELISA with calibration against standard curves and LC–MS/MS with internal standard normalization and predefined calibration ranges. Moreover, LC-MS/MS peptide analyses were performed in an independent university laboratory without access to clinical or microbiological data.

## Results

### Patient characteristics

The bacterial CAP cohort included *Streptococcus pneumoniae* (19%), *Mycoplasma pneumoniae* (22%), and *Klebsiella pneumoniae* (11%) as the most frequently identified pathogens. Viral CAP cases were predominantly caused by influenza A (22%) and rhinovirus/enterovirus (31%), followed by SARS-CoV-2 (11%), human metapneumovirus (11%), respiratory syncytial virus (4.4%), parainfluenza viruses (types 1, 3, and 4; 2.2%, 11%, and 4.4%, respectively), and adenovirus (2.2%) (Supplementary Table 1). Patients with viral CAP were older than those with bacterial CAP (median 75 vs. 69 years, *p* = 0.029) and had a higher proportion of males (67% vs. 36%, *p* = 0.008) (Table 1). Cardiovascular disease was more prevalent in viral CAP (76% vs. 36%, *p* = 0.001), whereas autoimmune (4.4% vs. 19%, *p* = 0.070) and malignant diseases (13% vs. 33%, *p* = 0.058) were less frequent, although not reaching statistical significance. No significant differences in disease severity were observed based on CRB-65 ≥ 2 (24% vs. 39%, *p* = 0.226). However, patients with bacterial CAP more frequently required oxygen therapy during hospitalization (50% vs. 11%, *p* < 0.001). Length of hospital stay was longer in viral CAP (median 10 vs. 6 days, *p* = 0.001). No differences were observed in fever prevalence (64% vs. 61%, *p* = 0.819) or survival (89% vs. 86%, *p* = 0.745) (Table 1).

**Table 1:** Clinical characteristics and outcomes of patients with bacterial and viral CAP

	Bacterial CAP^1^	Viral CAP^1^	Overall^1^	*p*-value^2^
No. of patients	*n* = 36	*n* = 45	*n* = 81	
Demographics				
Age, years	69 (59, 75)	75 (63, 81)	74 (62, 78)	0.029
Male sex	13 (36%)	30 (67%)	43 (53%)	0.008
Current smoker	7 (19%)	4 (8.9%)	11 (14%)	0.203
Comorbidities				
Chronic obstructive pulmonary disease (COPD)	10 (28%)	10 (22%)	20 (25%)	0.611
Cardiovascular disease	13 (36%)	34 (76%)	47 (58%)	0.001
Autoimmune disease	7 (19%)	2 (4.4%)	9 (11%)	0.070
Malignancy	12 (33%)	6 (13%)	18 (22%)	0.058
Disease severity				
CRB-65 score ≥ 2	14 (39%)	11 (24%)	25 (31%)	0.226
Oxygen requirement	18 (50%)	5 (11%)	23 (28%)	< 0.001
Therapies				
Amoxicillin clavulanate	4 (11%)	4 (8.9%)	8 (9.9%)	1.000
Piperacillin/ tazobactam	15 (42%)	11 (24%)	26 (32%)	0.099
Azithromycin	22 (61%)	13 (29%)	35 (43%)	0.004
Ampicillin/ sulbactam	22 (61%)	29 (64%)	51 (63%)	0.758
Clinical course				
Length of hospital stay, days	6 (5, 10)	10 (8, 14)	9 (6, 13)	0.001
Duration of therapies, days	7.5 (5, 11.5)	5 (3, 6)	7 (5, 10)	0.039
CRP (mg/l) at admission	131 (54, 218)	83 (49, 165)	96 (49, 169)	0.188
Fever (> 38 °C)	22 (61%)	29 (64%)	51 (63%)	0.819
Survival (In-hospital)	31 (86%)	40 (89%)	71 (88%)	0.745

^1^ Median (Q1, Q3); n (%), ^2^ Wilcoxon rank sum test; Fisher’s exact test

### Alpha-1 antitrypsin (AAT) levels and association with CAP etiology

**Fig. 1:**
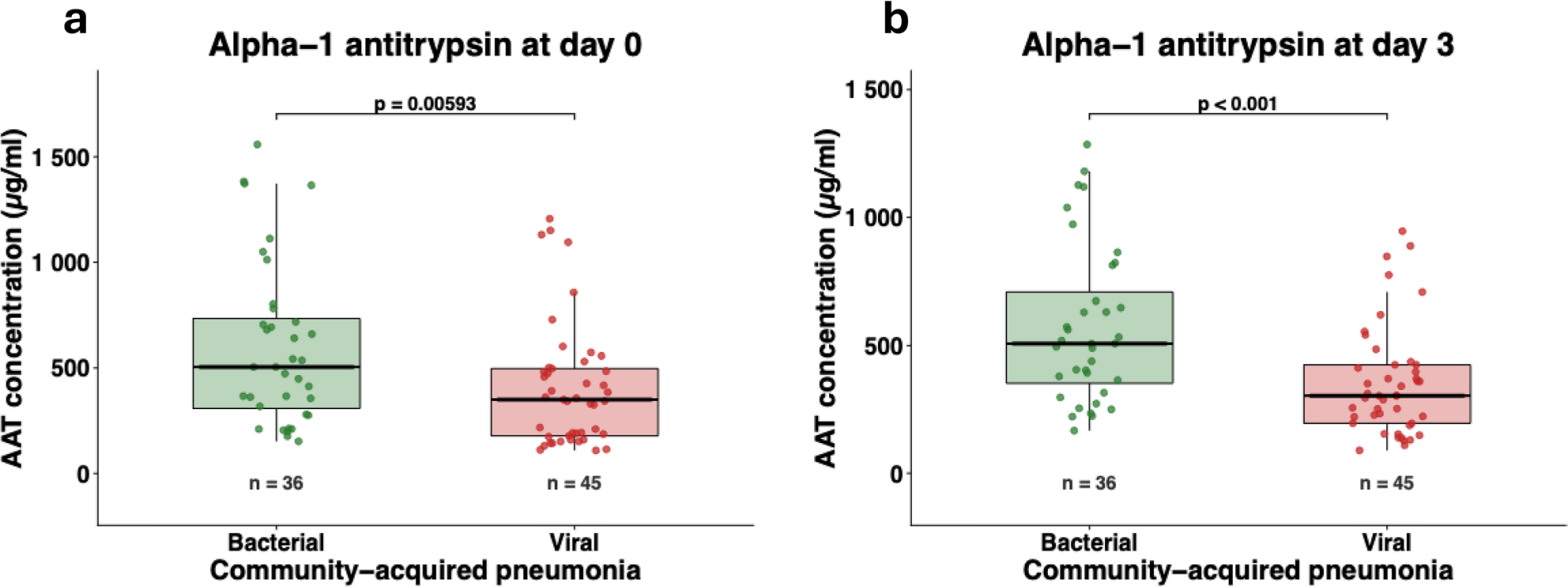
Serum AAT concentrations (µg/ml) in patients with bacterial (*n* = 36) and viral (*n* = 45) CAP at (**a**) hospital admission (day 0; *p* = 0.006) and (**b**) day 3 (*p* < 0.001)

**Fig. 2:**
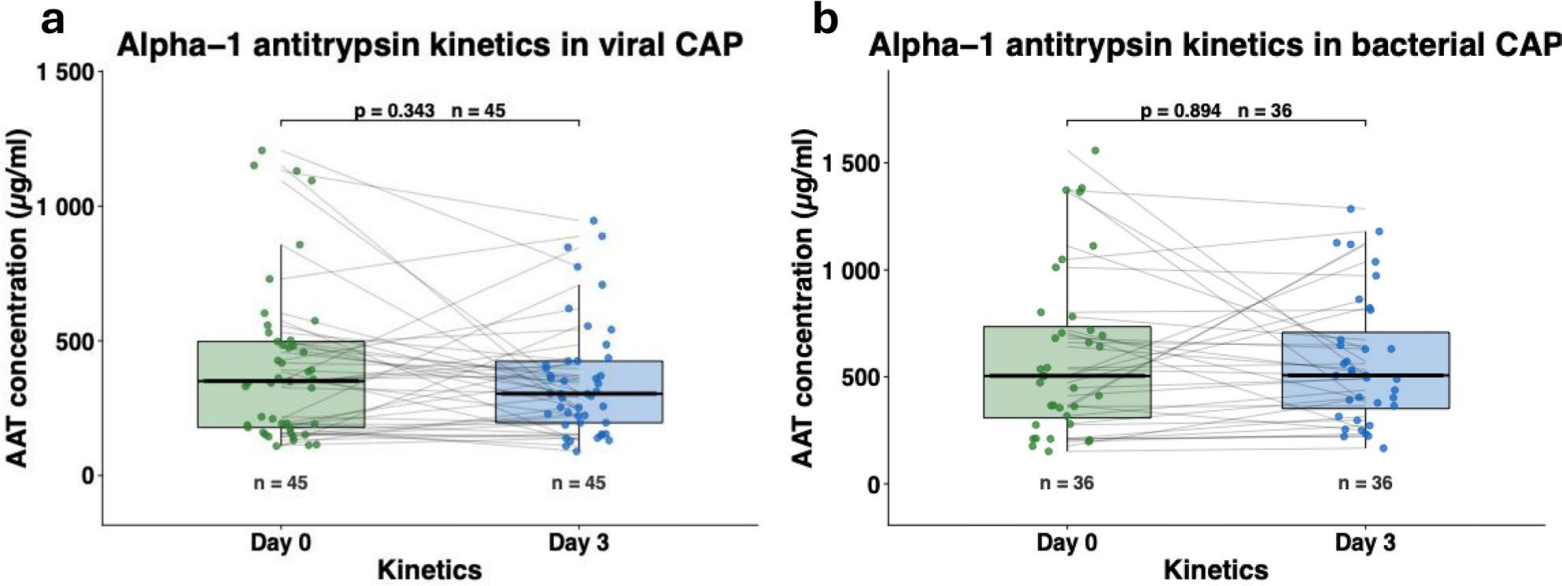
Longitudinal serum alpha-1 antitrypsin concentrations (µg/ml) from day 0 to day 3 in (**a**) viral CAP (*n* = 45; *p* = 0.343) and (**b**) bacterial CAP (*n* = 36; *p* = 0.894)

Serum AAT levels were significantly higher in bacterial compared with viral CAP at admission (day 0, *p* = 0.006) and day 3 (*p* < 0.001) (Fig. 1). No significant changes over time were observed within either group (bacterial: *p* = 0.894; viral: *p* = 0.343) (Fig. 2).

### Alpha-1 antitrypsin (AAT) association with CAP etiology

In univariate logistic regression, leukocyte count showed the strongest association with CAP etiology (OR 1.120, 95% CI 1.024–1.220, *p* = 0.009), followed by AAT levels at admission (OR 1.002, 95% CI 1.000–1.003, *p* = 0.017) and age (OR 0.958, 95% CI 0.930–0.990, *p* = 0.011), whereas CRP was not significantly associated (*p* = 0.236). AAT levels at day 3 also remained associated with CAP etiology (OR 1.003, 95% CI 1.001–1.005, *p* = 0.001). In ROC analysis, leukocyte count demonstrated the highest discriminatory performance at day 0 (AUC 0.696, 95% CI 0.573–0.810), followed by AAT (AUC 0.679, 95% CI 0.560–0.788) and age (AUC 0.642, 95% CI 0.517–0.755), while CRP performed poorly (AUC 0.586, 95% CI 0.463–0.705). In multivariable analysis, leukocyte count and AAT levels at admission remained independently associated with CAP etiology (OR 1.117, 95% CI 1.024–1.219, *p* = 0.013; OR 1.002, 95% CI 1.000–1.003, *p* = 0.021, respectively), whereas CRP showed no independent association (*p* = 0.818) (Table 2).

**Table 2:** Multivariable logistic regression: levels of AAT and CRP, and leukocyte counts at admission (day 0)

	Odds Ratio	95% CI	*p*-value
AAT	1.002	1.000-1.003	0.021
CRP	0.999	0.995–1.004	0.818
Leukocytes	1.117	1.024–1.219	0.013

**Table 3:** Multivariable logistic regression: AAT, age, leukocyte counts at day 0

	Odds Ratio	95% CI	*p*-value
AAT	1.001	1.000-1.003	0.056
Age	0.963	0.930–0.997	0.032
Leukocytes	1.118	1.023–1.221	0.014

Abbreviations: AAT, alpha1-antitrypsin; CI, confidence interval

**Fig. 3:**
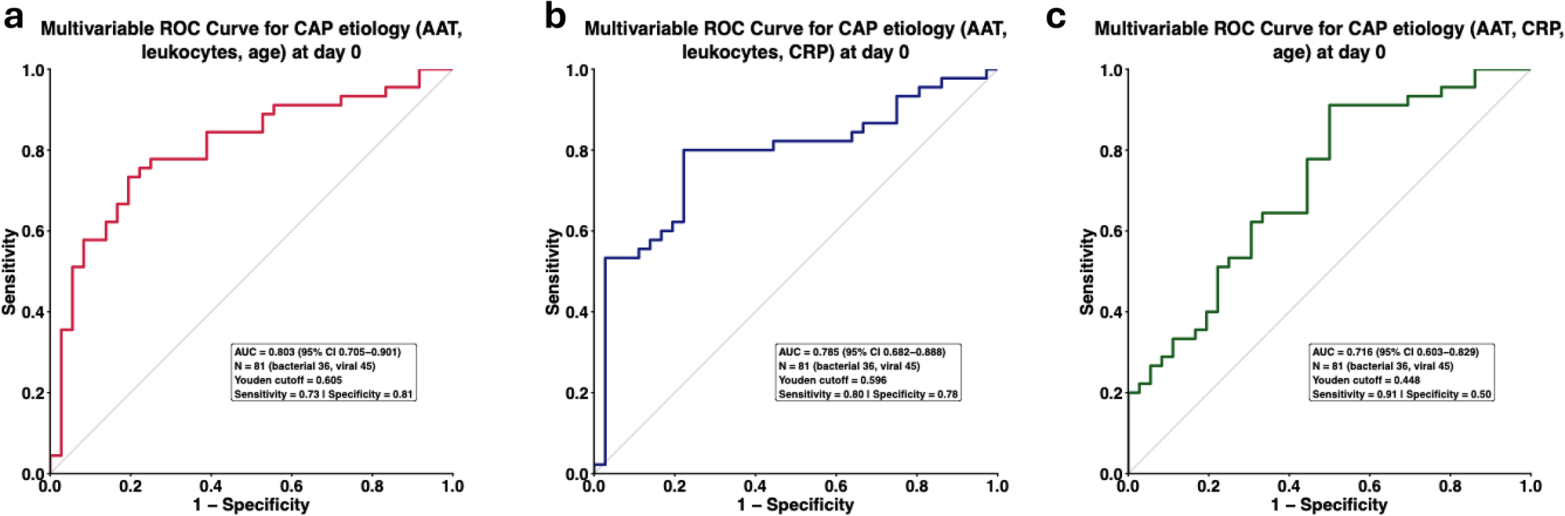
Multivariable ROC analyses for discrimination of CAP etiology (bacterial *n* = 36, viral *n* = 45) using combined models of (**a**) AAT, leukocyte count and age (AUC 0.803, 95% CI 0.705–0.901); (**b**) AAT, leukocyte count and CRP (AUC 0.785, 95% CI 0.682–0.888); and (**c**) AAT, CRP and age (AUC 0.716, 95% CI 0.603–0.829)

In a model including AAT, CRP, and age, AAT remained non-significant but with a similar effect estimate (OR 1.001, 95% CI 1.000–1.003, p=0.077), whereas CRP showed no independent association (OR 1.002, 95% CI 0.997–1.006, p=0.450). Age remained independently associated with CAP etiology (OR 0.963, 95% CI 0.931–0.996, p=0.027). Multivariable ROC analyses confirmed these findings, with the highest discriminatory performance observed for the combination of AAT, age, and leukocyte count (AUC 0.803, 95% CI 0.705–0.901). Inclusion of CRP did not improve model performance, with similar or lower AUCs for models including AAT, CRP, and leukocyte count (AUC 0.785, 95% CI 0.682–0.888) or AAT, CRP, and age (AUC 0.716, 95% CI 0.603–0.829) (Fig. 3a–c).

To address potential circularity in case definition, a sensitivity analysis restricted to microbiologically confirmed bacterial CAP (*n* = 19) versus viral CAP (*n* = 45) was performed, excluding 17 clinically defined bacterial cases. The difference in AAT levels between groups remained highly significant and showed a larger effect size in the restricted cohort (median 681.2 vs. 350.2 µg/ml, *p* < 0.001; Cohen’s d increased from 0.58 to 1.03). In multivariable analysis, AAT (OR 1.002 per µg/ml, 95% CI 1.000–1.004, *p* = 0.016) and age (OR 0.961, 95% CI 0.921–0.998, *p* = 0.047) remained independently associated with CAP etiology, whereas leukocyte count was no longer significant (*p* = 0.323).

To assess potential confounding by atypical pathogens, biomarker profiles were compared between patients with typical bacterial infection (*Streptococcus pneumoniae*,* Klebsiella pneumoniae*; *n* = 11) and atypical infection (*Mycoplasma pneumoniae*; *n* = 8). Despite the small subgroup sizes, no significant differences were observed at day 0 for AAT (681.2 vs. 624.5 µg/ml, *p* = 0.836), CRP (*p* = 0.591), or leukocyte count (*p* = 0.483). Age differed significantly between groups (74.0 vs. 35.5 years, *p* = 0.002), consistent with the younger age of patients with *Mycoplasma pneumoniae* infection.

### Levels of C-terminal AAT peptides in patients with CAP

Among the analyzed peptides, C37 levels were significantly higher in bacterial than viral CAP at admission (day 0, *p* = 0.010), while C36 showed a trend toward higher levels in bacterial CAP (*p* = 0.075) (Fig. S1a, b). No significant differences were observed for C40 or C42 at day 0 (*p* = 0.395 and *p* = 0.153, respectively) (Table 4, Fig. S1c, d). At day 3, C37 again showed a trend toward higher levels in bacterial CAP (*p* = 0.076), whereas no between-group differences were detected for C36, C40, or C42 (Fig. S2a–d).

**Table 4:** Comparison of peptide levels (*µM*) between bacterial and viral CAP at admission (day 0) and day 3

Peptides, (µM)	Bacterial CAP^1^ *n* = 36	Viral CAP^1^ *n* = 45	*p*-value^2^
C36 (day 0)	0.26 (0.19, 0.38)	0.23 (0.15, 0.32)	0.075
C36 (day 3)	0.26 (0.18, 0.3)	0.19 (0.15, 0.33)	0.427
C37 (day 0)	0.06 (0.04, 0.07)	0.04 (0.03, 0.05)	0.01
C37 (day 3)	0.05 (0.03, 0.08)	0.04 (0.03, 0.06)	0.076
C40 (day 0)	0.01 (0, 0.01)	0.01 (0.01, 0.01)	0.395
C40 (day 3)	0.01 (0.01, 0.01)	0.01 (0.01, 0.01)	0.213
C42 (day 0)	0.19 (0.15, 0.25)	0.16 (0.13, 0.23)	0.153
C42 (day 3)	0.2 (0.13, 0.24)	0.17 (0.11, 0.23)	0.161

^1^Median (IQR), ^2^Mann-Whitney-U-Test

**Fig. 4:**
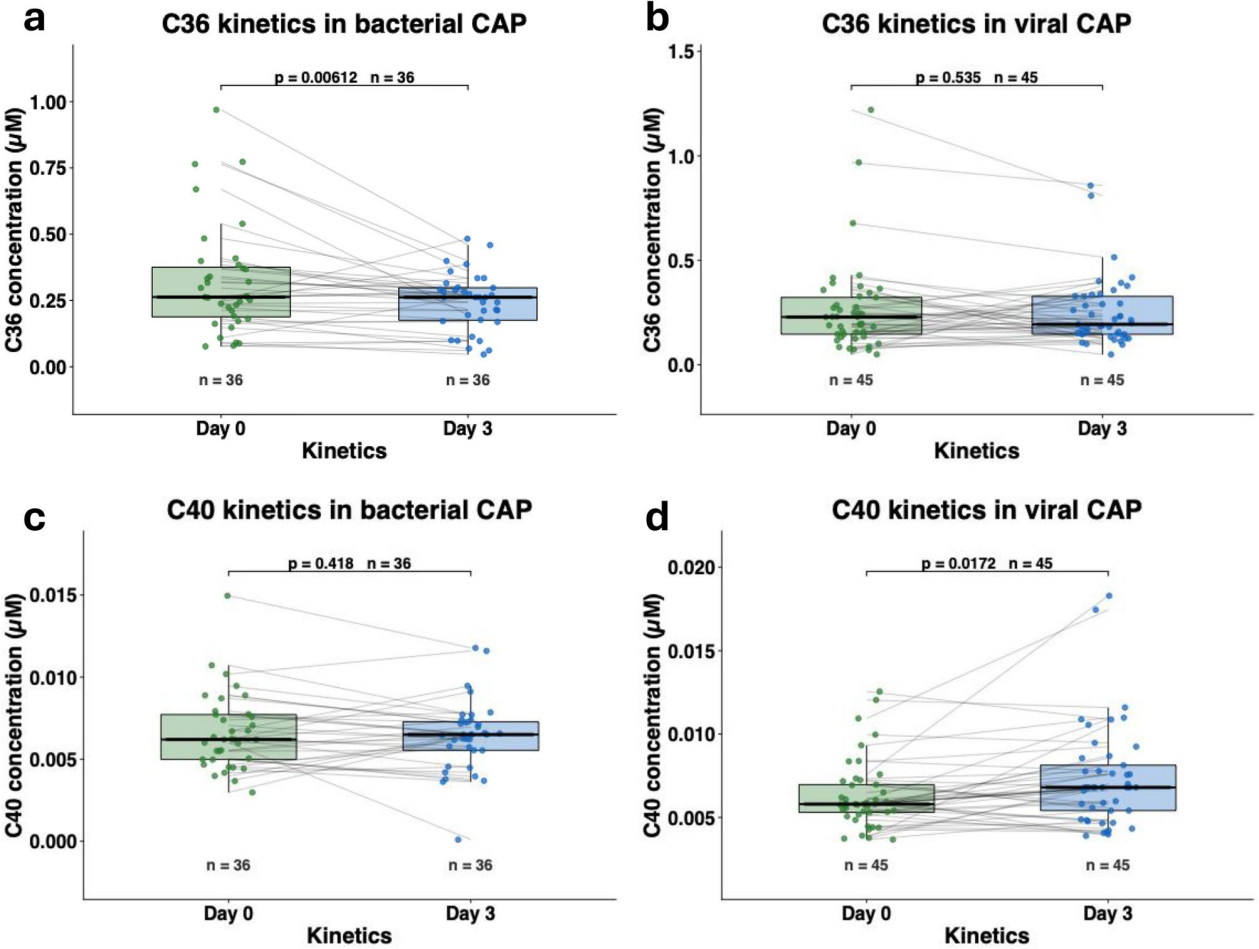
Temporal dynamics (day 0 → day 3) of C36 (µM) in (**a**) bacterial (*n* = 36; *p* = 0.006) and (**b**) viral CAP (*n* = 45; not significant), and of C40 (µM) in (**c**) bacterial (*n* = 36; not significant) and (**d**) viral CAP (*n* = 45; *p* = 0.017)

Longitudinally, in bacterial CAP, only C36 decreased significantly from day 0 to day 3 (*p* = 0.006), while C37, C40, and C42 remained stable. In viral CAP, C40 increased significantly over time (*p* = 0.017), whereas no significant changes were observed for C36, C37, or C42 (Fig. 4a–d; Fig. S3).

### Correlations between C-terminal peptides and AAT, and peptide-to-AAT ratios in patients with CAP

We first assessed correlations between individual C-terminal peptides and AAT in bacterial and viral CAP at both time points. At day 0, no significant correlations were observed in viral CAP. In bacterial CAP, C37 (ρ = 0.41, *p* = 0.012) and C42 (ρ = 0.47, *p* = 0.004) correlated significantly with AAT levels, while C40 showed a borderline association (ρ = 0.32, *p* = 0.053) and C36 showed no correlation. By day 3, these associations were largely absent, except for a newly observed correlation between AAT and C37 in viral CAP (*p* = 0.022) (Fig. S4–S5).

**Fig. 5:**
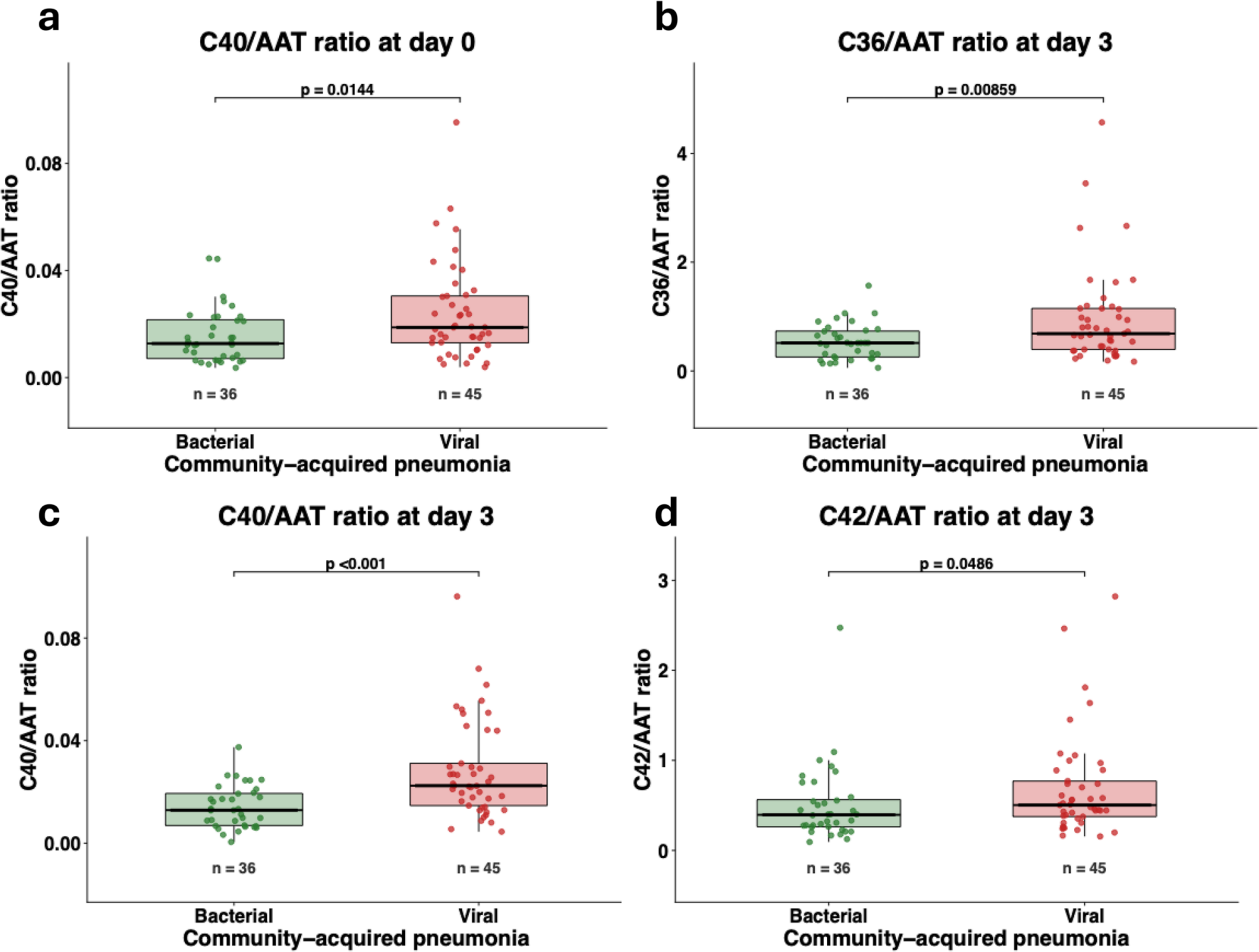
Comparison of C-terminal peptide/AAT ratios (dimensionless, ×1000) between bacterial (*n* = 36) and viral (*n* = 45) CAP: (**a**) C40/AAT ratio at day 0 (*p* = 0.014), (**b**) C36/AAT ratio at day 3 (*p* = 0.009), (**c**) C40/AAT ratio at day 3 (*p* < 0.001), and (**d**) C42/AAT ratio at day 3 (*p* = 0.049)

To distinguish peptide generation from differences in circulating AAT levels, peptide-to-AAT ratios were calculated. At day 0, only the C40/AAT ratio was significantly higher in viral compared with bacterial CAP (*p* = 0.014) (Fig. 5a), while no differences were observed for C36/AAT (*p* = 0.645), C37/AAT (*p* = 0.665), or C42/AAT (*p* = 0.285). At day 3, viral CAP showed significantly higher C36/AAT (*p* = 0.0086), C40/AAT (*p* < 0.001), and C42/AAT (*p* = 0.049) ratios, whereas the C37/AAT ratio did not differ between groups (*p* = 0.211) (Fig. 5b–d). Longitudinally, no significant within-group changes were observed, except for a borderline decrease in the C36/AAT ratio in bacterial CAP (*p* = 0.056).

**Fig. 6:**
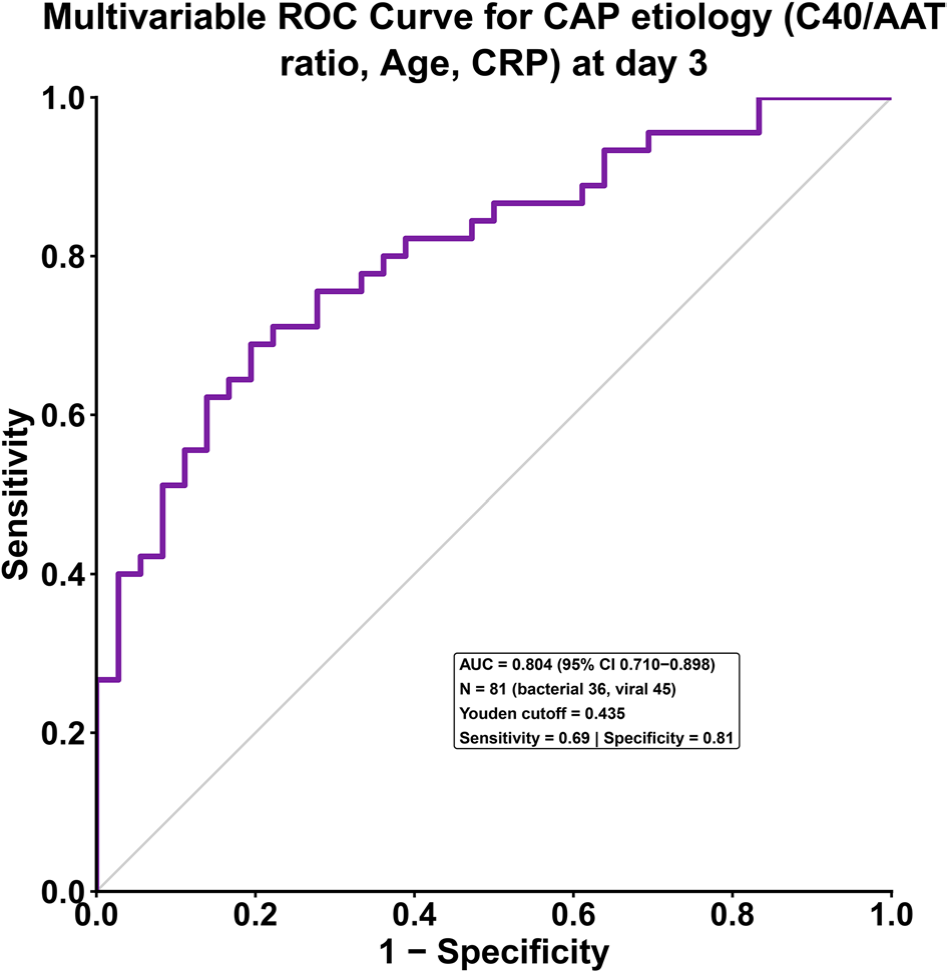
Multivariable ROC analysis of the C40/AAT ratio, CRP and age at day 3 for discrimination of CAP etiology (bacterial *n* = 36, viral *n* = 45; AUC 0.804, 95% CI 0.702–0.891)

In univariate logistic regression, at day 0 only the C40/AAT ratio was significantly associated with CAP etiology (OR 0.412, 95% CI 0.177–0.816, *p* = 0.022), and this association persisted at day 3 (OR 0.161, 95% CI 0.049–0.410, *p* < 0.001). At day 3 the C36/AAT ratio also showed a significant association (OR 0.972, 95% CI 0.947–0.992, *p* = 0.016; Table 5). In multivariable analysis including the C40/AAT ratio (day 3), age, and CRP, both the ratio and age remained independently associated with CAP etiology (Table 6), achieving discrimination comparable to the model based on AAT alone (AUC 0.804, 95% CI 0.702–0.891; Fig. 6).

**Table 5:** Univariate logistic regression analysis of the C-terminal peptide/ AAT ratios

Ratios at day 0	Odds Ratio	95% CI	*p*-value
C36/AAT	0.992	0.980–1.003	0.189
C37/AAT	0.981	0.908–1.053	0.594
C40/AAT	0.412	0.177–0.816	0.022
C42/AAT	0.984	0.963–1.001	0.094
C36/AAT day 3	0.972	0.947–0.992	0.016
C37/AAT day 3	0.940	0.861–1.015	0.134
C40/AAT day 3	0.161	0.049–0.410	< 0.001
C42/AAT day 3	0.984	0.960–1.002	0.121

**Table 6:** Multivariable logistic regression: C40/AAT, CRP, and age at day 3

	Odds Ratio	95% CI	*p*-value
C40/AAT	0.131	0.032–0.389	0.001
CRP	1.003	0.998–1.010	0.265
Age	0.962	0.924–0.996	0.043

### Correlations of AAT and its peptides with clinical parameters of CAP patients

**Fig. 7:**
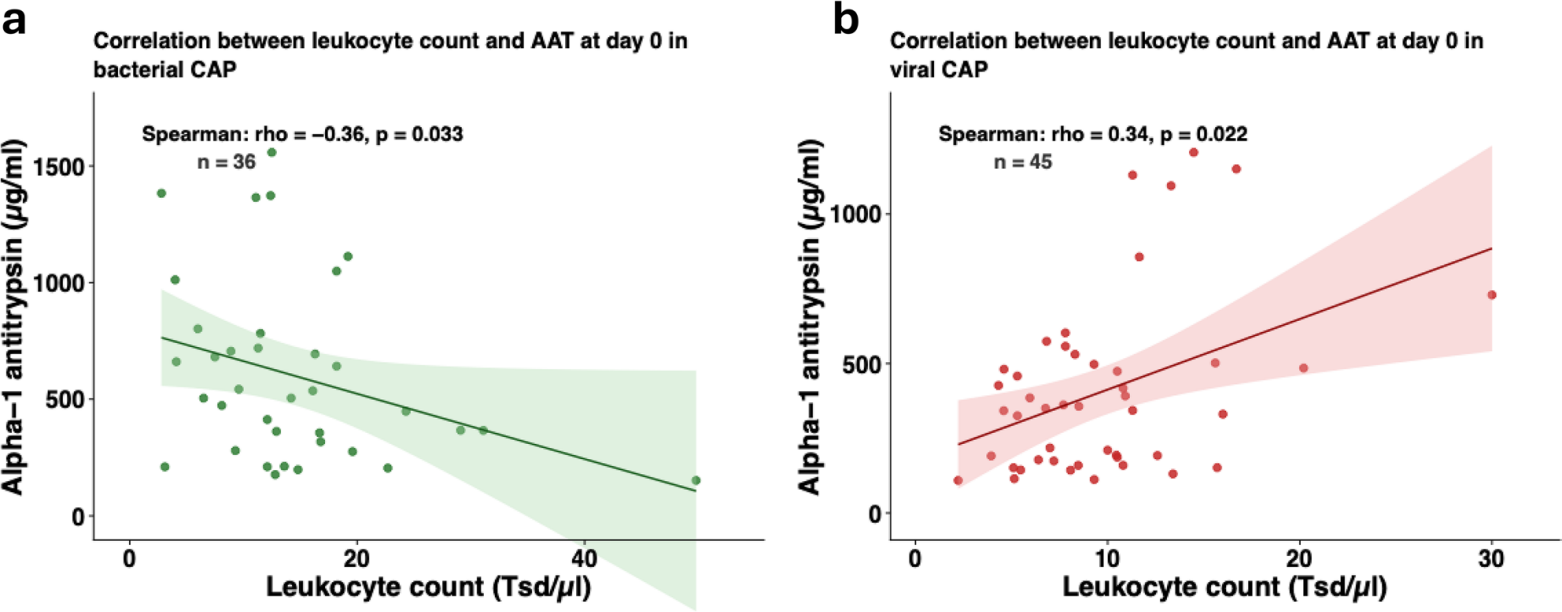
Spearman correlation between total leukocyte count and serum AAT at admission (day 0) in (**a**) bacterial CAP (*n* = 36; ρ = -0.36, *p* = 0.033) and (**b**) viral CAP (*n* = 45; ρ = 0.34, *p* = 0.022)

At day 0, AAT correlated with CRP in bacterial CAP (ρ = 0.36, *p* = 0.032), but not in viral CAP (Fig. S6). Similarly, C36, C37, and C40 correlated with CRP only in bacterial CAP, whereas C42 correlated with CRP in both bacterial and viral CAP (Fig. S7). AAT showed divergent correlations with leukocyte count, displaying a negative correlation in bacterial CAP (ρ=−0.36, *p* = 0.033) and a positive correlation in viral CAP (ρ = 0.34, *p* = 0.022) (Fig. 7). No significant correlations were observed between C-terminal peptides and leukocyte counts.

### Patient age-related associations with AAT levels

**Fig. 8:**
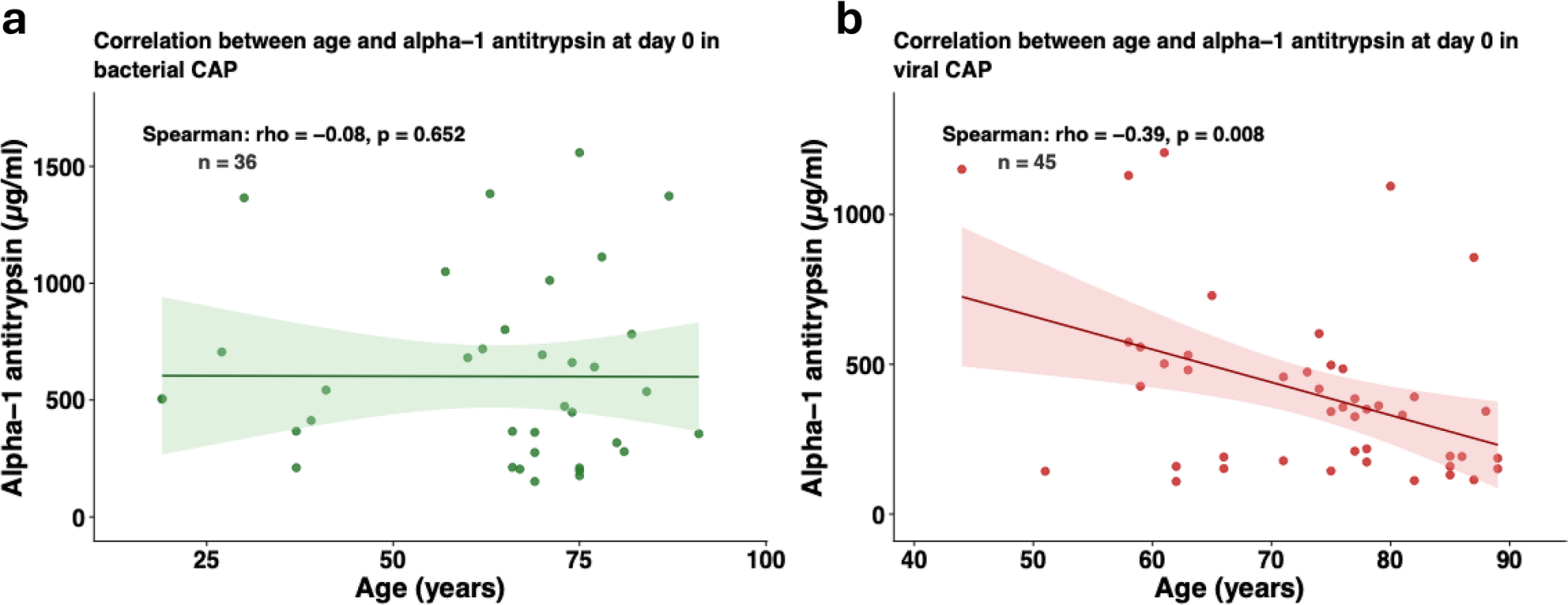
Spearman correlation between serum AAT at day 0 and patient age in (**a**) bacterial CAP (*n* = 36; *p* = 0.652) and (**b**) viral CAP (*n* = 45; ρ = -0.39, *p* = 0.008)

At day 0, AAT levels were inversely correlated with patient age in viral CAP (ρ=−0.39, *p* = 0.008), but not in bacterial CAP (*p* = 0.652). Multivariable linear regression including an interaction term confirmed that the relationship between age and AAT levels at day 0 differed significantly by CAP etiology (interaction *p* = 0.046). Specifically, older patients with viral CAP had lower AAT levels, whereas no such association was observed in bacterial CAP. No significant associations were found between peptides and age in either group (Fig. 8).

### Discussion

CAP triggers a heterogeneous host immune response that differs substantially between bacterial and viral infections [31, 32]. These differences are reflected in distinct systemic inflammatory and proteolytic responses [12, 33]. Despite these latter, reliable etiological discrimination remains a major clinical challenge. In routine practice, bacterial and viral CAP are often present with overlapping clinical features, while microbiological confirmation is frequently incomplete or delayed, resulting in diagnostic uncertainty. Current biomarkers, including CRP and other routine inflammatory parameters, offer only limited diagnostic performance. Even multimarker approaches generally achieve only moderate accuracy, with reported AUCs of approximately 0.7–0.8 [16, 34–37]. In addition, co-infections and suboptimal pathogen detection further complicate etiological assignments in real-world settings. These limitations highlight the need for improved host-response biomarkers that more directly reflect infection-specific immune and proteolytic processes. In this context, we investigated whether circulating AAT and its proteolytically generated C-terminal peptides could serve as discriminative biomarkers for bacterial versus viral CAP.

Like CRP, AAT is an acute-phase protein whose hepatic synthesis is primarily driven by IL-6 signaling [35]. Elevated IL-6 levels are a hallmark of bacterial pneumonia and correlate with disease severity [36], consistent with reports of higher AAT concentrations in pediatric patients with bacterial compared with viral CAP [36]. In adults, the AAT/IL-10 ratio has also been proposed as a predictor of bacterial pneumonia, with values ≥ 65 showing discriminatory potential independent of disease severity [7]. Furthermore, AAT concentrations have been shown to correlate with IL-6 and to be elevated in CAP compared with healthy controls, suggesting an association with Gram-positive infections [16]. However, it remains unclear whether circulating AAT levels alone differ between bacterial and viral CAP in adults.

In this study, we demonstrate that serum AAT levels are significantly higher in adults with bacterial compared with viral CAP. In addition, specific AAT-derived C-terminal peptides show differential associations with disease etiology. Together, these findings support AAT as a host-response biomarker in CAP and suggest that its proteolytic fragments provide complementary, mechanism-linked information reflecting infection-specific inflammatory activity. Importantly, a model combining AAT, leukocyte counts, and age showed improved discrimination of CAP etiologies. While AAT reflects systemic inflammatory activation, leukocyte counts provide complementary information on the cellular immune response, particularly neutrophil predominance in bacterial infection. Notably, AAT levels were inversely associated with age in viral CAP, with lower levels observed in older patients at admission. This may reflect age-related changes in immune responses, including impaired interferon signaling, a key pathway in antiviral immunity [37, 38]. Consequently, older individuals may exhibit a blunted acute-phase response to viral infection.

As a matter of fact, adding CRP did not improve model performance, likely reflecting the partial overlap between CRP and AAT as acute-phase reactants, while also suggesting that AAT captures additional aspects of the host response beyond general systemic inflammation. Beyond its role as an inflammatory marker, AAT is a functional regulator of protease activity and an immune modulator, thereby linking systemic immune activation with local proteolytic processes in the lung [39]. This dual functionality may explain the superior discriminatory performance of AAT compared with CRP.

Another notable observation is that serum AAT levels did not change significantly between day 0 and day 3 in either bacterial or viral CAP. This likely reflects the slower kinetics of AAT compared with other acute-phase reactants, such as CRP. Accordingly, early in-hospital fluctuations in AAT appear limited, supporting its role as a relatively stable marker of systemic inflammation [40].

Recent studies have highlighted the importance of host-response signatures that integrate both inflammatory and proteolytic pathways in CAP [34, 41]. In this context, AAT may reflect both the magnitude of the acute-phase response and the underlying protease-antiprotease balance, which are particularly pronounced in bacterial infections. Beyond total AAT levels, our data demonstrates in vivo proteolytic processing of AAT during pneumonia, resulting in detectable C-terminal peptides (C36, C37, C40, and C42) in serum. Among these, C37 was significantly elevated in bacterial CAP at admission, with C36 showing a similar trend. These AAT-derived peptides may arise not only from host proteases but also from pathogen-derived proteolytic activity [21, 42]. For example, pseudolysin from *Pseudomonas aeruginosa* generates C37 and C40 fragments of AAT [43], while streptococcal serine proteases such as HtrA may also cleave AAT [44], potentially during protease–antiprotease interactions involving AAT [45]. Together, these mechanisms link infection-driven protease activity to the generation of circulating AAT-derived peptide signatures.

As discussed above, CAP is associated with increased host protease activity and circulating matrix metalloproteases have been linked to CAP severity [46, 47]. Transcriptomic studies indicate distinct regulation of protease networks between bacterial and viral CAP, with bacterial infection characterized by enrichment of innate immunity and extracellular matrix remodeling proteases, whereas viral CAP is associated with increased expression of apoptosis-related proteases [48]. In this context, the high level of host-derived proteolytic activity may mask more subtle contributions from pathogen-specific proteases, particularly when both generate overlapping AAT-derived peptides. In contrast, peptide species preferentially generated by pathogen-associated proteases are less influenced by this background host proteolysis and may therefore offer higher diagnostic specificity. Accordingly, pathogen-associated peptide signatures may represent promising biomarker candidates in CAP and warrant further translational investigation.

To assess whether AAT-derived peptides reflect differential proteolytic processing rather than variation in total AAT levels, peptide concentrations were normalized to circulating AAT levels. This approach reduces confounding due to variability in total AAT levels. While absolute peptide concentrations showed only modest differences at admission (day 0), the C40/AAT ratio was significantly higher in viral CAP and remained elevated at day 3. In addition, C36/AAT and C42/AAT ratios were increased in viral CAP at day 3, suggesting time-dependent differences in proteolytic processing. Overall, normalization to AAT revealed alterations in the protease–antiprotease balance that were not apparent from absolute peptide levels alone. Among all analyzed peptides, the C40/AAT ratio most consistently discriminated viral CAP. Notably, only a limited number of proteases have been implicated in generating the C40 fragment from AAT, with cathepsin L (CTSL) being the best-characterized host-derived enzyme [49]. CTSL is a lysosomal cysteine protease that can be released during cellular stress and has been implicated in antiviral responses and epithelial cell injury [50], as well as in modulation of adaptive immunity [44, 51–53]. This may provide a potential explanation for the higher C40/AAT ratio observed in viral CAP, where epithelial injury and intracellular protease activation are more pronounced. Thus, specific peptide/AAT ratios, such as C40/AAT, may reflect distinct proteolytic pathways and could serve as more sensitive indicators of pathogen-specific host responses than absolute peptide concentrations alone.

Correlation analyses further revealed distinct patterns between bacterial and viral CAP. In bacterial CAP, AAT correlated with several peptides as well as CRP, consistent with a coordinated acute-phase and protease-driven response. In contrast, these associations were largely absent in viral CAP, suggesting a different or less tightly coupled inflammatory and proteolytic milieu. Remarkably, AAT showed opposite correlations with leukocyte counts in bacterial versus viral CAP potentially reflecting differences in immune cell dynamics between infection types. In bacterial CAP, the negative correlation suggests a partial dissociation between the hepatic acute-phase response and neutrophil-driven inflammation. In viral CAP, the positive correlation may indicate a more coordinated relationship between systemic inflammatory signalling and leukocyte dynamics. Overall, these findings suggest that bacterial and viral CAP differ not only in the magnitude but also in the coordination of systemic immune responses.

Notably, most correlations were weak to moderate (ρ ≈ 0.3–0.5), with only the associations between bacterial peptides and CRP reaching moderate strength. These findings suggest that AAT and its peptides reflect only part of the systemic inflammatory response and are more likely to represent etiology-dependent acute-phase and proteolytic activity than tightly coupled quantitative relationships. As these analyses are exploratory, they require validation in independent cohorts. Another important consideration is bacterial–viral coinfection, which is common in clinical practice and may elicit a mixed host response. Because the present study was designed to compare well-defined bacterial and viral CAP as distinct etiological groups, coinfections were not specifically evaluated. Whether AAT and its C-terminal peptides retain their discriminatory performance in this more complex clinical setting remains to be determined in larger, more heterogeneous validation cohorts.

The biological relevance of AAT-derived peptides is increasingly recognized. Previous studies have shown that C-terminal fragments of AAT express immunomodulatory, protease-regulatory, and antimicrobial effects [27, 30, 54] and may contribute to host defense mechanisms [55, 56]. Our findings extend this concept by demonstrating the in vivo generation of these peptides in patients with CAP. The lack of association between AAT-derived peptides and age further suggests that peptide generation is driven primarily by infection-related proteolytic activity rather than age-dependent baseline differences.

The present study focused on bacterial and viral CAP, as no cases of fungal or mycobacterial pneumonia were identified in either cohort. Whether distinct AAT-derived peptide signatures also characterize these less common CAP etiologies remains to be determined and represents an important direction for future translational research.

This study has several limitations. The cohort size was relatively small, and some peptide associations did not reach statistical significance, which limits firm conclusions about their diagnostic value. In addition, bacterial CAP was partly defined by clinical criteria without viral testing, which may have led to some misclassification. A further limitation concerns the assessment of neutrophil recruitment. Although bacterial CAP is typically characterized by neutrophil-dominated inflammation, differential neutrophil counts were not systematically recorded in a standardized format across the entire cohort and therefore could not be included as a quantitative variable in the statistical analyses. The bacterial group was mainly composed of *Mycoplasma pneumoniae* infections, which likely contributed to the younger age and shorter hospital stay in this group. Although multivariable analysis was used, some residual confounding by age between typical and atypical bacterial infections cannot be fully excluded. A further limitation is the study design. The bacterial CAP cohort was recruited prospectively at a single center, while the viral CAP cohort came from the multicenter CAPNETZ archive. Even though the same inclusion criteria were applied, small differences in sample collection and processing between prospective and archived samples cannot be ruled out.

Because this is an observational study, mechanistic conclusions regarding peptide generation and function cannot be drawn. A further limitation is potential pre-analytical variation, as viral CAP samples were retrieved from a biobank (− 80 °C, median storage 3.5 years), whereas bacterial CAP samples were processed prospectively and stored at − 20 °C, introducing systematic differences in storage conditions. Although a storage-related effect cannot be fully excluded, both AAT and its C-terminal peptides have been reported to be stable under long-term freezing and repeated freeze–thaw cycles [26, 27], making major degradation unlikely. In addition, several key findings were based on peptide-to-AAT ratios, which are internally normalised and less affected by absolute concentration differences. Nevertheless, validation in uniformly processed prospective cohorts is warranted.

## Conclusions

In adults with CAP, serum AAT levels were significantly higher in bacterial than viral disease and independently associated with etiological classification. A simple model combining AAT, leukocyte count, and age discriminated bacterial from viral CAP with good performance and outperformed CRP-based approaches, while CRP added no predictive value. We further characterized circulating AAT-derived C-terminal peptides in CAP, demonstrating in vivo proteolytic processing and identifying etiology-specific signatures, including an increased C40/AAT ratio in viral CAP. These findings suggest that AAT fragmentation reflects distinct pathogen-associated protease activity and immune response patterns. Together, intact AAT and its derived peptides provide complementary information on the systemic inflammatory and proteolytic host response. These markers represent promising CRP-independent candidates for CAP etiological discrimination and warrant validation in larger, prospectively collected, standardized cohorts.

## Supplementary Information

Below is the link to the electronic supplementary material.

Supplementary Material 1

## Data Availability

The data supporting the findings of this study contain potentially identifying patient information and are therefore not publicly available due to ethical and privacy restrictions imposed by the approving ethics committees (Hannover Medical School, No. 11910-BO-S-2025 and No. 301–2008). Supplementary figures referenced in the manuscript are provided as Supplementary Material. Aggregated source data underlying the main figures (e.g., group-level medians and interquartile ranges) can be provided by the corresponding author upon request. In exceptional cases, de-identified individual patient-level data may be made available from the corresponding author upon reasonable request, subject to a data sharing agreement and approval by the Ethics Committee of Hannover Medical School and, where applicable, the CAPNETZ Study Group.

## Declarations

### Generative AI and AI-assisted technologies in the writing process

During the preparation of this work, the authors used OpenAI ChatGPT (San Francisco, CA, USA) to assist with paraphrasing selected text passages, language refinement, and brainstorming of ideas during early manuscript drafting. In addition, Anthropic Claude (San Francisco, CA, USA) was used to support manuscript organization and to provide suggestions during refinement. The authors carefully reviewed and verified the accuracy, comprehensiveness, and impartiality of all AI-assisted output, including independent verification of any sources, as AI-generated references can be incorrect or fabricated. All material produced with AI assistance was thoroughly edited and adapted to ensure that the manuscript represents the authors' authentic and original contribution and reflects their own analysis, interpretation, insights, and ideas. The authors reviewed the terms and conditions of the AI tools used to ensure that data privacy, intellectual property, and other rights were safeguarded throughout the manuscript preparation process. The authors take full responsibility for the content of the publication.

## Abbreviations

AAT: Alpha-1 antitrypsin
AUC: Area under the (ROC) curve
C36, C37, C40, C42: C-terminal alpha-1 antitrypsin-derived peptides
CAP: Community-acquired pneumonia
COPD: Chronic obstructive pulmonary disease
CRP: C-reactive protein
CTSL: Cathepsin L
ELISA: Enzyme-linked immunosorbent assay
LC-MS/MS: Liquid chromatography-tandem mass spectrometry
OR: Odds ratio
ROC: Receiver operating characteristic
