# Supplementary material for: Circulating alpha-1 antitrypsin and its c-terminal peptides differentiate bacterial from viral community-acquired pneumonia
Source: J Transl Med. 2026 Jul 20;24:944. doi: 10.1186/s12967-026-08651-8 (PMC13390320; doi:10.1186/s12967-026-08651-8)
Supplement: Supplementary file 1 — Supplementary Material 1 [file 12967_2026_8651_MOESM1_ESM.docx]

**Supplementary Materials**

**Supplementary Table 1.** Distribution of identified etiological agents in patients with community-acquired pneumonia (CAP).

|  | Bacterial CAP^1^ | Viral CAP^1^ | Overall^1^ |
| --- | --- | --- | --- |
| No. of patients | n = 36 | n = 45 | n = 81 |
| Bacterial pathogens |  |  |  |
| Streptococcus pneumoniae | 7 (19%) | 0 (0%) | 7 (8.6%) |
| Klebsiella pneumoniae | 4 (11%) | 0 (0%) | 4 (4.9%) |
| Mycoplasma pneumoniae | 8 (22%) | 0 (0%) | 8 (9.9%) |
| Viral pathogens |  |  |  |
| SARS-CoV-2 | 0 (0%) | 5 (11%) | 5 (6.2%) |
| Influenza A  Respiratory syncytial virus (RSV) | 0 (0%)  0 (0%) | 10 (22%)  2 (4.4%) | 10 (12%)  2 (2.5%) |
| Human metapneumovirus | 0 (0%) | 5 (11%) | 5 (6.2%) |
| Parainfluenza virus type 1 | 0 (0%) | 1 (2.2%) | 1 (1.2%) |
| Parainfluenza virus type 3 | 0 (0%) | 5 (11%) | 5 (6.2%) |
| Parainfluenza virus type 4 | 0 (0%) | 2 (4.4%) | 2 (2.5%) |
| Rhinovirus or enterovirus | 0 (0%) | 14 (31%) | 14 (17%) |
| Adenovirus | 0 (0%) | 1 (2.2%) | 1 (1.2%) |

^1^ n (%)

**Fig. S1.** Serum concentrations of C-terminal AAT peptides (µM) in bacterial (n = 36) and viral (n = 45) CAP at day 0: (a) C36 (p = 0.075), (b) C37 (p = 0.010), (c) C40 (p = 0.395), (d) C42 (p = 0.153).

**Fig. S2.** Serum concentrations of C-terminal AAT peptides (µM) in bacterial (n = 36) and viral (n = 45) CAP at day 3: (a) C36 (p = 0.427), (b) C37 (p = 0.076), (c) C40 (p = 0.213), (d) C42 (p = 0.161).

**Fig. S3.** Temporal dynamics (day 0 to day 3) of C-terminal AAT peptides (µM): C37 in (a) bacterial (n = 36; p = 0.61) and (b) viral CAP (n = 45; p = 0.557), and C42 in (c) bacterial (n = 36; p = 0.621) and (d) viral CAP (n = 45; p = 0.857).

**Fig. S4.** Spearman correlations between serum AAT (µg/ml) and C-terminal peptides (µM) at day 0: AAT vs C36 in (a) bacterial (n = 36; ρ = 0.24, p = 0.15) and (b) viral CAP (n = 45; ρ = 0.19, p = 0.22); AAT vs C37 in (c) bacterial (n = 36; ρ = 0.41, p = 0.012) and (d) viral CAP (n = 45; ρ = 0.19, p = 0.205)

**Fig. S5.** Spearman correlations between serum AAT (µg/ml) and C-terminal peptides (µM) at day 0: AAT vs C40 in (a) bacterial (n = 36; ρ = 0.32, p = 0.053) and (b) viral CAP (n = 45; ρ = 0.12, p = 0.428); AAT vs C42 in (c) bacterial (n = 36; ρ = 0.47, p = 0.004) and (d) viral CAP (n = 45; ρ = 0.13, p = 0.395).

**Fig. S6.** Spearman correlation between serum AAT (µg/ml) and CRP (mg/l) at day 0 in (a) bacterial CAP (n = 36; ρ = 0.36, p = 0.032) and (b) viral CAP (n = 45; ρ = 0.06, p = 0.699).

**Fig. S7.** Spearman correlations in bacterial CAP (n = 36) between C-terminal peptides (µM) and CRP (mg/l) at day 0: (a) C36 (ρ = 0.62, p <0.001), (b) C37 (ρ = 0.38, p = 0.022), (c) C40 (ρ = 0.46, p = 0.005), (d) C42 (ρ = 0.68, p <0.001).
